# Markers of Intestinal Permeability and Inflammation in Enterally Fed Children with Cerebral Palsy

**DOI:** 10.3390/nu16152447

**Published:** 2024-07-27

**Authors:** Dorota Mickiewicz-Góra, Katarzyna Sznurkowska, Karolina Skonieczna-Żydecka, Arleta Drozd, Anna Borkowska, Maciej Zagierski, Joanna Troch, Agnieszka Szlagatys-Sidorkiewicz

**Affiliations:** 1 Department of Paediatrics, Gastroenterology, Allergology & Paediatric Nutrition, Medical University of Gdansk, 80-803 Gdansk, Poland; anna.borkowska@gumed.edu.pl (A.B.); maciej.zagierski@gumed.edu.pl (M.Z.); joanna.troch@o2.pl (J.T.); agnieszka.szlagatys-sidorkiewicz@gumed.edu.pl (A.S.-S.); 2 Department of Biochemical Science, Pomeranian Medical University in Szczecin, 70-204 Szczecin, Poland; karolina.skonieczna.zydecka@pum.edu.pl; 3 Department of Human Nutrition and Metabolomics, Pomeranian Medical University in Szczecin, 70-204 Szczecin, Poland; arleta.drozd@pum.edu.pl

**Keywords:** cerebral palsy, inflammation, permeability, calprotectin, zonulin, IFABP

## Abstract

Cerebral palsy (CP) results in non-progressive damage to the central nervous system, leading to functional disorders of the gastrointestinal tract and requiring enteral nutrition via gastrostomy in some patients. The aim of the study was to assess the impact of enteral nutrition on intestinal inflammation expressed by stool calprotectin and intestinal permeability determined by fecal zonulin and IFABP, and to determine whether CP affects these parameters. The study group consisted of 30 children with CP, fed enterally (Cerebral Palsy Enteral Nutrition—CPEN), and two reference groups: 24 children with CP, fed orally with a standard diet (CPC—Cerebral Palsy Controls) and 24 healthy children (HC—healthy controls). The differences between these groups and between the combined CP groups (CPG and CPEN + CPC) and HC were analyzed. Fecal zonulin, calprotectin, and intestinal fatty acid-binding protein 2 (IFABP2) levels were determined by ELISA. The concentrations of fecal calprotectin and zonulin were significantly higher in the CPEN group than in the CPC group (*p* = 0.012, *p* = 0.025). When comparing the CPG (*n* = 53) with the HC group (*n* = 24), statistically significant differences were observed for calprotectin (*p* = 0.000018, higher in the CPG) and IFABP (*p* = 0.021, higher in HC). Enteral nutrition was associated in our cohort with increased fecal calprotectin and zonulin. Children with cerebral palsy presented with increased fecal calprotectin but not increased intestinal permeability expressed by stool zonulin.

## 1. Introduction

Cerebral palsy is a set of heterogenous symptoms resulting in non-progressive impairment of the central nervous system, which may appear in fetal life, infancy, and subsequently up to 2 years old [1]. It is one of the most common causes of disability in children [2]. The impairment of the nervous system leads to a number of negative consequences for other body systems, including the digestive system. Gastrointestinal tract disorders, such as dysphagia, might arise, necessitating enteral nutrition via a gastrostomy tube.

The intestinal barrier is a functional multilayer structure separating the intestinal lumen from the interior of the body, creating an external physical barrier and an internal functional barrier. It consists of mechanical, humoral, immunological, muscular, and neurological components, which are shown in Table 1 [3,4].

The intestinal epithelial barrier functions to minimize the passage of pathogens, microbial factors, and toxins from the lumen into the gut tissue and then into the bloodstream and may even cause neuroinflammation and behavioral changes [5,6].

Many factors may adversely affect the functions of the intestinal barrier, including lifestyle, dietary factors (e.g., Western-style, energy dense diet), epithelial damage, and alterations of the mucus layer. This can result in the translocation of the content of the gut lumen to further layers of the intestinal wall (in the following sequence: the mucosa, submucosa, muscularis propria, and serosa). This process is known as “intestinal permeability” [4]. Increased intestinal permeability has been also found in various diseases, such as inflammatory bowel disease, neoplasia, irritable bowel syndrome, and other functional gastrointestinal diseases, autoimmune diseases (e.g., celiac disease), intestinal hypoperfusion, allergies, malnutrition, metabolic diseases associated with obesity (e.g., cardiovascular diseases, diabetes type 1 and 2, NASH), and as a consequence of taking non-steroidal anti-inflammatory drugs [3,4]. Several markers of intestinal permeability exist, including zonulin and IFABP2, which were investigated in this study.

Zonulin is a protein responsible for regulating intestinal permeability by altering the structure and functions of the tight junctions between the cells of the digestive track wall. The tight junctions control the movement of fluids, macromolecules, and leukocytes between the intestinal lumen and the bloodstream [7,8]. Zonulin is actively engaged in the intestinal innate immune response [7,9] and several factors, including diet (e.g., apple pectin, colostrum bovinum, and vitamins A and D) may lower zonulin levels [10,11]. It is assumed that zonulin activation may be a defense mechanism to bacterial infections, preventing bacteria from adhering to and colonizing the small intestine, because increased secretion of this protein was observed after exposure to various strains of bacteria (both pathogenic and non-pathogenic) [10,12]. Apart from the intestine, zonulin may be involved in other tissues, including brain and heart, including fetal and adult tissues. Zonulin levels can be measured in serum and stool samples [13].

Intestinal fatty acid binding protein (IFABP/FABP-2) is another, less commonly recognized biomarker of intestinal barrier dysfunction. It is found in the cytoplasm of enterocytes from the duodenum to the ileum, and also in colonic mucosa (2–3% of the enterocyte’s cytoplasmic protein mass) [14,15,16,17,18]. It is considered to be a novel biomarker of tissue damage in the upper gastrointestinal tract (a marker of enterocyte injury) [14] and can also indicate intestinal necrosis [17]. Its role is to uptake fatty acids into cells and transport them to organelles, to deliver fatty acids to metabolic pathways, and to protect cell membranes and enzymes from high concentrations of free fatty acids [16]. The IFABP concentration is typically low under normal conditions, but it increases in the circulation when intestinal epithelial cells are damaged [14]. Data about IFABP are still limited, but it appears that it is suitable as a noninvasive diagnostic tool for celiac disease and Crohn’s disease [15]. Cut-off levels have not yet been agreed upon. 

Fecal calprotectin, widely known as a noninvasive marker of gut inflammation, is linked with intestinal permeability [19,20]. Calprotectin comprises 60% of the cytosolic protein in neutrophiles and is also found, in lower proportions, in monocytes and macrophages. It is associated with inflammatory cytokines activity, and can be found in serum, urine, saliva, cerebrospinal fluid, synovial fluid, amniotic fluid, and stool samples [19,20]. Much research is ongoing on other factors that may influence calprotectin levels, including drugs, diet, and type of feeding at the beginning of life (breastfeeding vs. formula feeding) [20]. Its levels are predominantly increased in inflammatory bowel disease (and ulcerative colitis and Crohn’s disease) [21], which is helpful in differentiating between inflammatory bowel disease (IBD) and irritable bowel syndrome (IBS) [19]. It has high sensitivity, but low specificity in children with IBD [20]. Cut-off levels have not been agreed upon, with proposed cut-offs including 50 µg/g, 100 µg/g, and 200 µg/g. Therefore, 100 µg/g is frequently accepted as a balanced value with moderate levels of sensitivity and specificity for the diagnosis of Crohn’s disease [22].

Children with cerebral palsy are likely to suffer from malnutrition, which has been reported, depending on the source, in 29% to 46% of children with CP. Feeding and nutritional problems resulting from dysphagia, gastroesophageal reflux (GER), and oral motor dysfunction are more likely to occur in CP children, as are spillage, vomiting, delayed gastric emptying, and constipation. Enteral nutrition (EN) is, thus, one of feeding options indicated in CP when nutrient and energy requirements exceed oral intake and cannot be covered by regular food intake [23]. Gastrostomy placement effectively improves patients’ nutritional status, feeding time, and caregiver satisfaction [24]. EN is also used in other diseases, especially in Crohn’s disease, in which it seems to have anti-inflammatory potential, and plays essential role in the treatment [25,26,27].

We aimed to assess whether enteral nutrition impacts intestinal inflammation as expressed by stool calprotectin and intestinal permeability determined by zonulin and IFABP in children with CP, and to verify if the disease itself influences these parameters. Assessing the correlation between calprotectin and the parameters of intestinal permeability was an additional objective of the study.

## 2. Materials and Methods

### 2.1. Participants

The study was approved by the Independent Bioethics Committee of the Medical University of Gdansk (no NKBBN/324/2017, 18 October 2017). The study group consisted of 30 children (F = 17, M = 13) who were diagnosed with cerebral palsy and received enteral nutrition (Cerebral Palsy Enteral Nutrition (CPEN)) via gastrostomy and were patients of the Outpatient Nutrition Clinic of the Copernicus Hospital in Gdansk and the Department of Paediatrics, Gastroenterology, Allergology and Paediatric Nutrition at the Medical University of Gdansk. Two reference groups were enrolled in the study. The first reference group (Cerebral Palsy Controls (CPC)) consisted of 23 children (F = 8, M = 15) diagnosed with cerebral palsy and fed orally on a regular diet, while 24 healthy children (F = 11, M = 13) with no chronic disease and fed on a regular diet were enrolled in the second reference group (healthy controls (HC)). Thus, in total, 53 patients with CP and 24 healthy individuals participated in the study. The inclusion criteria for the study group were as follows: written caregiver consent, age under 18 years old, a diagnosis of cerebral palsy, enteral nutrition with commercial diets, no inflammatory diseases of gastrointestinal tract, and no antibiotic or probiotic therapy in the period of 3 months prior to the date of the stool sample collection. The reference group inclusion criteria included a written caregiver consent, age under 18 years old, diagnosed cerebral palsy, no diagnosed chronic inflammatory diseases of the gastrointestinal tract, oral nutrition with a regular diet, and no antibiotic or probiotic therapy in the period of 3 months before the date of the stool sample collection. The study group exclusion criteria were age above 18 years old, enteral nutrition with standard homemade diet (without commercial diets), inflammatory diseases of the gastrointestinal tract, and antibiotic or probiotic therapy in the period of 3 months preceding the date of the stool sample collection. The reference group’s exclusion criteria were age above 18 years old, inflammatory diseases of the gastrointestinal tract, and antibiotic or probiotic therapy in the period of 3 months preceding the date of the stool sample collection. The inclusion criteria are summarized in Table 2 below.

Characteristics of groups are presented in Table 3.

### 2.2. Procedures

After obtaining the medical history, basic anthropometric measurements (body weight and height) were carried out. Measurement of the body weight was carried out in the morning, on an empty stomach, using the weight function of the InBody 120 body composition analyzer (InBody Co., Ltd. South Korea Manufacturers, Seoul, Republic of Korea). The measurement was taken with the precision of 100 g. A stadiometer was used to measure the heigth for standing children, and an anthropometric measuring tape was used for children with a limited ability to maintain a standing position. The accuracy of recording the result was 1 mm. The length measurement of children with limb spasticity was challenging, because they had to lay down, and we had to take into account their body curvature. In order to calculate the average values, the measurements were taken three times. The calculation of BMI percentiles was performed, and the results were verified using the WHO Growth Standards in children under 5, and the Development Standards for Children and Adolescents aged 6–18 [28]. The standard deviations distribution (SD, z-score) was used to evaluate nutritional status [29]. The international GMFCS scale (Gross Motor Function Classification Scale) shown in Table 4 below was used to assess the gross motor skills of the patients. The GMFCS accurately portrays the gross motor abilities of children with CP at different ages. Scores were assessed for every patient with cerebral palsy in both the CPEN and CPC groups. The scale does not apply to healthy children [30].

### 2.3. Fecal Sample Collection

Prior to collecting the stool sample, the parents were trained in how to properly collect it. The stool samples were collected by the patients’ caregivers in a clean container to prevent contamination of the sample. After the stool sample was given to the examiner, it was immediately frozen at −80 °C. The samples were then transported on dry ice to the Department of Human Nutrition and Metabolomics, Pomeranian Medical University in Szczecin in Poland for testing.

### 2.4. Assessment of Investigated Parameters: Intestinal Barrier Markers and Calprotectin

The investigated parameters included zonulin (ZO), calprotectin (CLP), and intestinal fatty acid binding protein (IFABP). Stool samples required for the assessment were prepared according to the assay’s manufacturer’s instructions.

Fecal zonulin, calprotectin (Immundiagnostik AG, Bensheim, Germany, catalog numbers: K 5600 and K 6967) and intestinal fatty acid binding protein 2 (Cloud-Clone Corp., Wuhan, China, cat no. SEA559Hu) levels were determined using an enzyme-linked immunosorbent assay (ELISA) according to the manufacturer’s protocol. The absorbance was measured with a spectrophotometer (Sunrise, Tecan, Männedorf, Switzerland) at 450 nm. 

The concentration of calprotectin, zonulin, and IFABP were expressed in ng/mL. 

For the purpose of this study, the following intergroup differences in the investigated parameters were analyzed: CPEN and CPC, CPC and HC, and CPG (CPEN + CPC) and HC.

### 2.5. Statistical Analysis

The Shapiro–Wilk test was used to verify the normality of the distribution of continuous variables. Due to significant deviations from a normal distribution, median and ranges were used to describe continuous variables. Qualitative variables were reported using counts and percentages. Accordingly, the significance of intergroup differences was verified with the Mann–Whitney U test. The relationships between pairs of variables were analyzed on the basis of Spearman’s rank correlation coefficients. The results of all tests were considered significant at *p* < 0.05. All analyses were performed with the Statistica 12 software package (Stat Soft. Inc., Tulsa, OK, USA).

Spearman rank correlations were used for continuous variables. A two-sided *p*-value of 0.05 was considered the level of significance.

## 3. Results

**Figure 1:**
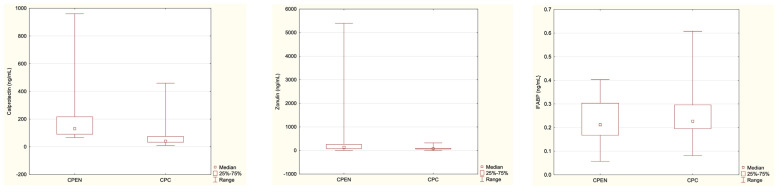
Differences in the investigated parameters between the CPEN and CPC groups, with medians (indicated by small red squares), 25–75% (indicated by large red rectangles), and ranges. CPEN—Cerebral Palsy Enteral Nutrition; CPC—Cerebral Palsy Controls.

The median level of fecal calprotectin was significantly higher in the CPEN than in the CPC group (*p* = 0.012) (Table 5, Figure 1).

The median concentration of zonulin was significantly higher in the CPEN than in the CPC group (*p* = 0.025) and in HC vs. CPC (Table 5, Figure 1).

No statistically significant differences were found between the CPEN and CPC groups in median levels of IFABP2 (*p* = 0.62) (Table 5, Figure 1).

Detailed numerical data, including median, range, mean, and SD and *p*-values, are shown in Table 5.

Among the CPEN patients, there were four samples with noticeably higher levels of fecal zonulin compared to the other samples (above 1000 ng/mL—5395.05 ng/mL, 2471.9 ng/mL, 1946.55 ng/mL, and 1430 ng/mL). There was also one HC patient with a very high zonulin level—1193.9 ng/mL. Among the CPC patients, there were no patients with a comparably high level of zonulin. An extremely high level of calprotectin in a stool sample was found in a patient from the CPEN group, at 960.60 μg/mL. This was a different patient than the three with the extremely high zonulin levels.

No statistically significant differences were found between concentrations of fecal calprotectin and IFABP in the CPC and HC groups (*p* = 0.07, *p* = 0.10, respectively). Zonulin levels were significantly lower in the CPC groups when compared with the HC group (*p* = 0.04) (Table 5).

**Figure 2:**
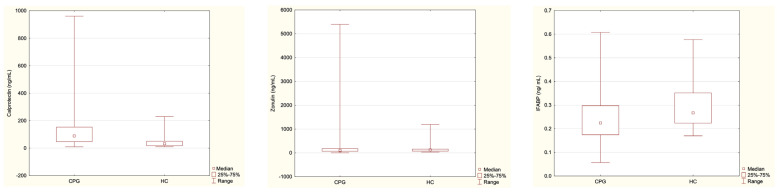
Differences in the investigated parameters between the CPG and HC groups, with medians (indicated by small red squares), 25–75% (indicated by large red rectangles), and ranges. CPG—Cerebral Palsy Group; HC—healthy controls.

Comparing children with cerebral palsy (CPG, i.e., CPEN and CPC together, *n* = 53) to healthy controls (HC, *n* = 24), statistically significant differences were observed for calprotectin (*p* = 0.000018, higher in the CPG) and IFABP (*p* = 0.021, higher in the HC) (Table 6 and Figure 2). Zonulin concentration did not differ significantly between cerebral palsy patients and healthy controls (*p* = 0.54) (Table 6, Figure 2).

No statistically significant correlations were found between zonulin, calprotectin, and IFABP levels and age, BMI, or GMFCS within the groups. No correlation was found between zonulin and calprotectin, zonulin and IFABP, and IFABP and calprotectin in the CPEN, CPC, or HC groups.

Percentages of children with normal and increased calprotectin levels are presented in Table 7.

Among the CPEN patients, there was no patient with a normal calprotectin level: in 80% (*n* = 24) of the patients’ calprotectin was between 50–250 ng/mL, in 13.33% (*n* = 4) of the patients’ calprotectin was between 250–600 ng/mL, and 6.67% (*n* = 2) of the patients had extremely high calprotectin levels, between 600 and 1000. Such a high level of calprotectin was not observed in any CPC or HC patients.

Percentages of children with normal and increased zonulin levels are presented in Table 8.

Among the CP group, there were 23.33% of patients with normal levels of zonulin; among the CPC group it was 26.09%, and in the HC group, it was 20.83%. In every group of patients, the majority of patients had increased levels of zonulin (above 60): 76.67% in the CPEN group, 73.91% in the CPC group, and 79.17% in the HC group.

## 4. Discussion

Intestinal permeability and inflammation are currently under extensive investigation, with the aim of exploring the relationship between various diseases and higher gut permeability and attempting to determine if it is caused by inflammation. To the best of our knowledge, our study is the first to assess calprotectin, zonulin, and IFABP levels in children with cerebral palsy fed enterally and it is also the only one investigating the impact of enteral nutrition on intestinal permeability.

Calprotectin as an inflammatory marker has high sensitivity but low specificity, with increased levels in stool samples found in numerous conditions, including inflammatory bowel disease [31], Helicobacter Pylori gastritis [32], microscopic colitis [33], polyps [19], necrotizing enterocolitis [34,35], celiac disease [35], acute enterocolitis [35], acute gastroenteritis [36], cow’s milk protein allergy [37], and even diseases not related to the digestive tract, such as septicemia, meningitis, pneumonia [38], juvenile idiopathic arthritis, and glomerulonephritis [34]. Evidence shows that calprotectin differentiates between functional gastrointestinal diseases and organic ones [36]. There are some studies concerning calprotectin in inflammatory bowel disease (IBD) in comparison to irritable bowel syndrome (IBS). According to Olender et al. (2012), a cutoff point of 100 µg/g, instead of 50 µg/g, should be considered—in the pediatric group, it has a higher specificity in the diagnosis of IBD [20]. According to Orfei et al. (2021), children with fecal calprotectin <600 μg/g without gastrointestinal symptoms (e.g., abdominal pain, diarrhea) are not likely to have IBD [39]. Using fecal calprotectin in pediatric gastroenterology has a big advantage: as a noninvasive marker, it helps to reduce unnecessary exposure to endoscopy [20,40,41]. Moreover, for a long time, it has been widely used in pediatric IBD as an imperfect but a relatively effective parameter for monitoring of the disease [25,26,42].

In our research, we found that children from the CPEN group had higher stool calprotectin levels than the patients in the CPC group. This result seems to be surprising in the light of the existing evidence showing the anti-inflammatory effects of enteral nutrition in Crohn’s disease. 

Enteral nutrition has been shown to cause not only reductions in calprotectin levels but also improvements in clinical, endoscopic, and mucosal changes in children with Crohn’s disease, and for this reason, it is used as a treatment option in mild to moderate Crohn’s disease. Evident reductions in calprotectin levels were, for example, described one month after the introduction of exclusive enteral nutrition [43,44]. Enteral nutrition exhibits a direct anti-inflammatory action, as evidenced by a reduction in inflammatory cytokines, and promotes mucosal healing even before visible nutritional benefits become apparent [45]. The efficacy of EN in decreasing stool calprotectin was even demonstrated for partial enteral nutrition in children with Crohn’s disease [26].

In our cohort, none of the patients from the CPEN group presented with a normal calprotectin level. On the other hand, the values of this parameter were moderately elevated in the majority of the patients and only two patients had levels above the cutoff point proposed for IBD [44,46]. It is challenging to account for the unexpected results of our study. One possible explanation can be the higher incidence of motility disorders noted in the CPEN group (Table 3). Functional gastrointestinal disorders, typical for cerebral palsy, can themselves have proinflammatory potential, as was shown by the study of Choi, who documented elevated calprotectin in children with irritable bowel syndrome [36]. We have to note that this result might simply be the consequence of a small study sample and should be verified in a larger cohort.

Comparison of stool calprotectin levels between all the patients with cerebral palsy (CPG = CPC + CPEN) and the healthy children revealed significant differences in our cohort. These results seem to confirm the notion of the proinflammatory potential of cerebral palsy postulated by some authors. According to Colson et al. (2013), who described the increased incidence of IBD in CP, this predisposition may result from treatment for CP and other comorbidities that may impact the function of the gastrointestinal tract, undefined nutritional deficiencies, or an altered intestinal environment predisposing to immune dysregulation [47]. As we have mentioned above, the role of impaired motor function associated with cerebral palsy could contribute to the increased calprotectin in cerebral palsy individuals in the presented study. We must note, however, that calprotectin in the CPC group was not significantly higher than in the HC group and was within the normal range in 60.87% patients. The difference observed between the whole cerebral palsy group and the healthy controls resulted predominantly from the high values observed in the CPEN group. Thus, our results do not align with the idea of the proinflammatory potential of CP as such.

As intestinal permeability is regarded as one of the hallmarks of gut inflammation, calprotectin and zonulin, the most widely described marker of intestinal integrity disfunction, are often investigated together [5,48].

The data concerning intestinal permeability in the pediatric population are very limited and none of the studies concern cerebral palsy. In the available data, we found higher fecal zonulin levels in children with the following diseases: IBD [25,49], pediatric nonalcoholic fatty liver disease fibrosis [50], celiac disease [51], rotavirus infection [52], but also with diseases not related to the gastrointestinal tract, such as glucose metabolism dysregulation, type 2 diabetes [53], and autism spectrum disorders [54]. In our research, we demonstrate that the median concentration of fecal zonulin was significantly higher in the CPEN group than in CPC group (*p* = 0.025). It seems important to draw attention to the fact that in the CPEN group, we found four extremely high values (5395.05 ng/mL, 2471.9 ng/mL, 1946.55 ng/mL, and 1430 ng/mL) and one such high value in the HC group (1193.9 ng/mL). In the available literature, the highest fecal zonulin levels were reported by Szymanska et al. (2022), with the range of 7.0–3854 ng/mL in children with Crohn’s disease. This study, demonstrating higher fecal zonulin and fecal calprotectin in IBD patients compared to healthy controls, concluded that zonulin may be an important marker of gut damage [49]. The results are, however, inconsistent and in another research, contrary to calprotectin, no significant results for zonulin were found in children with Crohn’s disease or ulcerative colitis [55]. 

In our study, we found the difference in zonulin levels between the CPEN and CPC groups. This result seems to be consistent with the result concerning calprotectin, which was also higher in CPEN compared to CPC patients. Moreover, contrary to our initial hypothesis, we did not find any correlation between zonulin and calprotectin, which could support the coherence of the obtained results. Furthermore, we did not find a difference in fecal zonulin between the whole CPG and HC groups, and, unexpectedly, we observed higher zonulin levels in the HC group than in the CPC group. These findings seem to show no impact of CP itself on intestinal permeability. How enteral nutrition could influence gut permeability remains unclear.

Another permeability indicator we investigated was IFABP, which is also regarded as a marker of tissue damage and necrosis of the enterocytes. It was previously reported in conditions such as Crohn’s disease, ulcerative colitis, bowel necrosis [18], and celiac disease [15]. It is very important to note that the available data concern only serum IFABP, not fecal levels of this parameter, so we do not have any comparative data for our results. To the best of our knowledge, this is the first study assessing IFABP levels in the stool. The results of existing publications (which are also scarce) concerning serum IFABP are inconsistent. While a study concerning children with celiac disease and another one on pediatric IBD reported increased levels of IFABP, other research noted decreased levels in adult patients with IBD [56,57]. Interestingly, even in IBD, none of the studies investigated IFABP in the stool.

In our study, we did not find statistically significant differences in fecal IFABP between the CPEN and CPC groups or CPC and HC groups. Unexpectedly, healthy controls had significantly higher values of fecal IFABP than cerebral palsy patients.

Considering the very limited publications and no other existing studies on fecal IFABP, further research in this field is necessary, especially as the search for noninvasive markers of intestinal damage is still ongoing.

## 5. Conclusions

Enteral nutrition was associated in our cohort with increased fecal calprotectin and zonulin.

Children with cerebral palsy presented with increased fecal calprotectin but not increased intestinal permeability expressed by stool zonulin.

Lower fecal IFABP was found in cerebral palsy. Further research on intestinal permeability in the context of inflammation is necessary in children with cerebral palsy.

## Figures and Tables

**Table 1:** Components of the intestinal barrier [3].

Type of Intestinal Barrier	Component
Mechanical	Mucus epithelial layer (tight junctions)
Humoral	Defensins, immunoglobulin A
Immunological	Lymphocytes, innate immune cells
Muscular	Smooth muscles
Neurological	Enteric nervous system

**Table 2:** Group inclusion criteria.

Group	Presence of Cerebral Palsy	Enteral Nutrition
CPEN	+	+
CPC	+	−
HC	−	−

CPEN—Cerebral Palsy Enteral Nutrition; CPC—Cerebral Palsy Controls; HC—healthy controls.

**Table 3:** Group characteristics.

Characteristics	CPEN (*n* = 30)	CPC (*n* = 23)	HC (*n* = 24)
Age	10.80 (±3.96)	8.96 (±4.38)	8.08 (±4.34)
Length/Height (cm)	131.18 (±16.65)	122.96 (±25.30)	127.56 (±29.31)
Body mass (kg)	22.92 (±5.95)	22.69 (±13.66)	32.30 (±21.90)
BMI	13.30 (±2.28)	13.93 (±3.30)	17.50 (±3.90)
GMFCS	4.767 (±0.50)	4.21 (±0.88)	NA
Dysphagia	56.67% (*n* = 17)	4.34% (*n* = 1)	0% (*n* = 0)
GERD	46.67% (*n* = 14)	21.73% (*n* = 5)	16.66% (*n* = 4)
Constipation	73.33% (*n* = 22)	26.08% (*n* = 6)	20.83% (*n* = 5)
Abdominal pain	30% (*n* = 9)	39.13% (*n* = 9)	16.66% (*n* = 4)

CPEN—Cerebral Palsy Enteral Nutrition; CPC—Cerebral Palsy Controls; HC—healthy controls; GMFCS—Gross Motor Function Classification Scale; NA—not applicable; GERD—gastroesophageal reflux disease.

**Table 4:** Gross Motor Function Classification Scale (GMFCS).

Level	Description
Level I	Walks without limitations
Level II	Walks with limitations
Level III	Walks with a handheld mobility device
Level IV	Requires physical assistance or uses powered mobility
Level V	Transported in a manual wheelchair in all settings

**Table 5:** Zonulin, calprotectin, and IFABP levels in the study and control groups.

Parameter	CPEN (*n* = 30)	CPC (*n* = 23)	HC (*n* = 24)
Calprotectin [ng/mL]	96.23	41.15	32.18
Median range	8.02–960.60	9.76–458.95	10.31–229.25
Mean ± SD	152.43 ± 202.54	84.39 ± 114.10	47.05 ± 50.84
*p*-value	***p* = 0.012**		
		*p* = 0.07	
Zonulin [ng/mL]	137.88	71.2	122.43
Median range	0.45–5395.05	3.25–322.75	32.85–1193.9
Mean ± SD	530.95 ± 1091.59	89.97 ± 67.34	176.41 ± 235.94
*p*-value	***p* = 0.0025**		
		***p* = 0.04**	
IFABP [ng/mL]	0.21	0.23	0.27
Median range	0.06–0.40	0.08–0.61	0.17–0.577
Mean ± SD	0.23 ± 0.09	0.25 ± 0.11	0.29 ± 0.10
*p*-value	*p* = 0.62		
		*p* = 0.10	

CPEN—Cerebral Palsy Enteral Nutrition; CPC—Cerebral Palsy Controls; HC—healthy controls; *p*-value—level of significance (statistically significant differences are bolded).

**Table 6:** Zonulin, calprotectin, and IFABP content in the cerebral palsy group and control group.

Parameter	CPG (*n* = 53)	HC (*n* = 24)
Calprotectin	57.72	32.18
Median range	8.02–960.59	10.31–229.25
Mean ± SD	122.88 ± 171.89	47.05 ± 50.84
*p*-value	***p* = 0.000018**	
Zonulin	93.25	122.43
Median range	0.45–5395.05	32.85–1193.9
Mean ± SD	339.58 ± 845.66	176.41 ± 235.94
*p*-value	*p* = 0.54	
IFABP	0.22	0.27
Median range	0.06–0.61	0.17–0.58
Mean ± SD	0.24 ± 0.1	0.29 ± 0.1
*p*-value	**0.021**	

CPG—Cerebral Palsy Group; HC—healthy controls; *p*-value—level of significance (statistically significant differences are bolded).

**Table 7:** Calprotectin levels in groups.

Range (ng/mL)	CPEN (*n* = 30)	CPC (*n* = 23)	HC (*n* = 24)
0–50	-	60.87% (*n* = 14)	75% (*n* = 18)
50–250	80% (*n* = 24)	30.43% (*n* = 7)	25% (*n* = 6)
250–600	13.33% (*n* = 4)	8.7% (*n* = 2)	-
600–1000	6.67% (*n* = 2)	-	-

CPEN—Cerebral Palsy Enteral Nutrition; CPC—Cerebral Palsy Controls; HC—healthy controls.

**Table 8:** Zonulin levels in groups.

Range (ng/mL)	CPEN (*n* = 30)	CPC (*n* = 23)	HC (*n* = 24)
0–60	23.33% (*n* = 7)	26.09% (*n* = 6)	20.83% (*n* = 5)
>60	76.67% (*n* = 23)	73.91% (*n* = 17)	79.17% (*n* = 19)

CPEN—Cerebral Palsy Enteral Nutrition; CPC—Cerebral Palsy Controls; HC—healthy controls.

## Data Availability

The data presented in this study are available on request from the corresponding author due to privacy of the patients.
